# Norovirus Infection in Harbor Porpoises

**DOI:** 10.3201/eid2301.161081

**Published:** 2017-01

**Authors:** Miranda de Graaf, Rogier Bodewes, Cornelis E. van Elk, Marco van de Bildt, Sarah Getu, Georgina I. Aron, Georges M.G.M. Verjans, Albert D.M.E. Osterhaus, Judith M.A. van den Brand, Thijs Kuiken, Marion P.G. Koopmans

**Affiliations:** Erasmus Medical Centre, Rotterdam, the Netherlands (M. de Graaf, R. Bodewes, C.E. van Elk, M. van de Bildt, S. Getu, G.I. Aron, G.M.G.M. Verjans, J.M.A. van den Brand, T. Kuiken, M.P.G. Koopmans);; Dolphinarium Harderwijk, Harderwijk, the Netherlands (C.E. van Elk);; SOS Dolphin Foundation, Harderwijk, the Netherlands (C.E. van Elk);; University of Veterinary Medicine, Hannover, Germany (A.D.M.E. Osterhaus)

**Keywords:** Norovirus, porpoise, Phocoena phocoena, genotype GI, marine mammals, intestine, viruses, zoonoses

## Abstract

A norovirus was detected in harbor porpoises, a previously unknown host for norovirus. This norovirus had low similarity to any known norovirus. Viral RNA was detected primarily in intestinal tissue, and specific serum antibodies were detected in 8 (24%) of 34 harbor porpoises from the North Sea.

Noroviruses have been detected in humans, cats, dogs, pigs, sheep, cattle, sea lions, rodents, and bats (*1*–*4*). In humans, norovirus is a leading cause of gastroenteritis (*1*). Seven different norovirus genogroups have been described for the norovirus genus (*1*) that can be further subdivided in ≈30 genotypes. Noroviruses comprise a single-strand positive-sense RNA genome that is divided into 3 open reading frames (ORFs). Recombination among different genotypes is frequently observed for human noroviruses, most commonly near the junction of ORF1 and ORF2, leading to a recommendation for multilocus genotyping (*5*). Surprisingly, recombinant human noroviruses regularly contain previously undetected ORF1 sequences, which raises questions about the reservoirs of these viruses.

Noroviruses can spread through the fecal–oral route, and sewage contamination in coastal environments can result in contamination of shellfish, such as oysters. Oysters filter several liters of seawater daily and contain histo-blood group antigens resembling those of humans. These antigens can be specifically bound by noroviruses, resulting in bioaccumulation (*6*); as a result, eating oysters is linked to foodborne norovirus outbreaks in humans (*7*). This mode of transmission, however, could expose humans to viruses from other animal reservoirs, such as marine mammals, and vice versa.

## The Study

A juvenile male harbor porpoise (*Phocoena phocoena*) ≈10.5 months of age was found alive on the coast of the Netherlands on May 10, 2012, and was transported to the SOS Dolphin Foundation (Harderwijk, the Netherlands) for rehabilitation (Technical Appendix). Important clinical signs at the rehabilitation center were anorexia, labored breathing, and disorientation. The animal showed no evidence of gastrointestinal disease, such as vomiting or diarrhea. Eight days after arrival in the rehabilitation center, the animal was euthanized because of the severity of clinical signs, and necropsy was performed according to standard procedures (*8*). The main pathology findings were bronchopneumonia associated with lungworm infection and encephalitis and hepatitis of unknown cause. The intestine did not show significant lesions macroscopically; microscopically, the enterocytes at the luminal surface of the intestine had sloughed into the lumen as a result of freeze–thaw artifact. The cells lining the intestinal crypts consisted of a mixture of enterocytes and mucus cells. The proportion of mucus cells increased progressively toward the end of the intestine. The lamina propria was infiltrated diffusely with a moderate number of lymphocytes, plasma cells, and eosinophils. This infiltrate was considered normal for this species.

**Figure 1:**
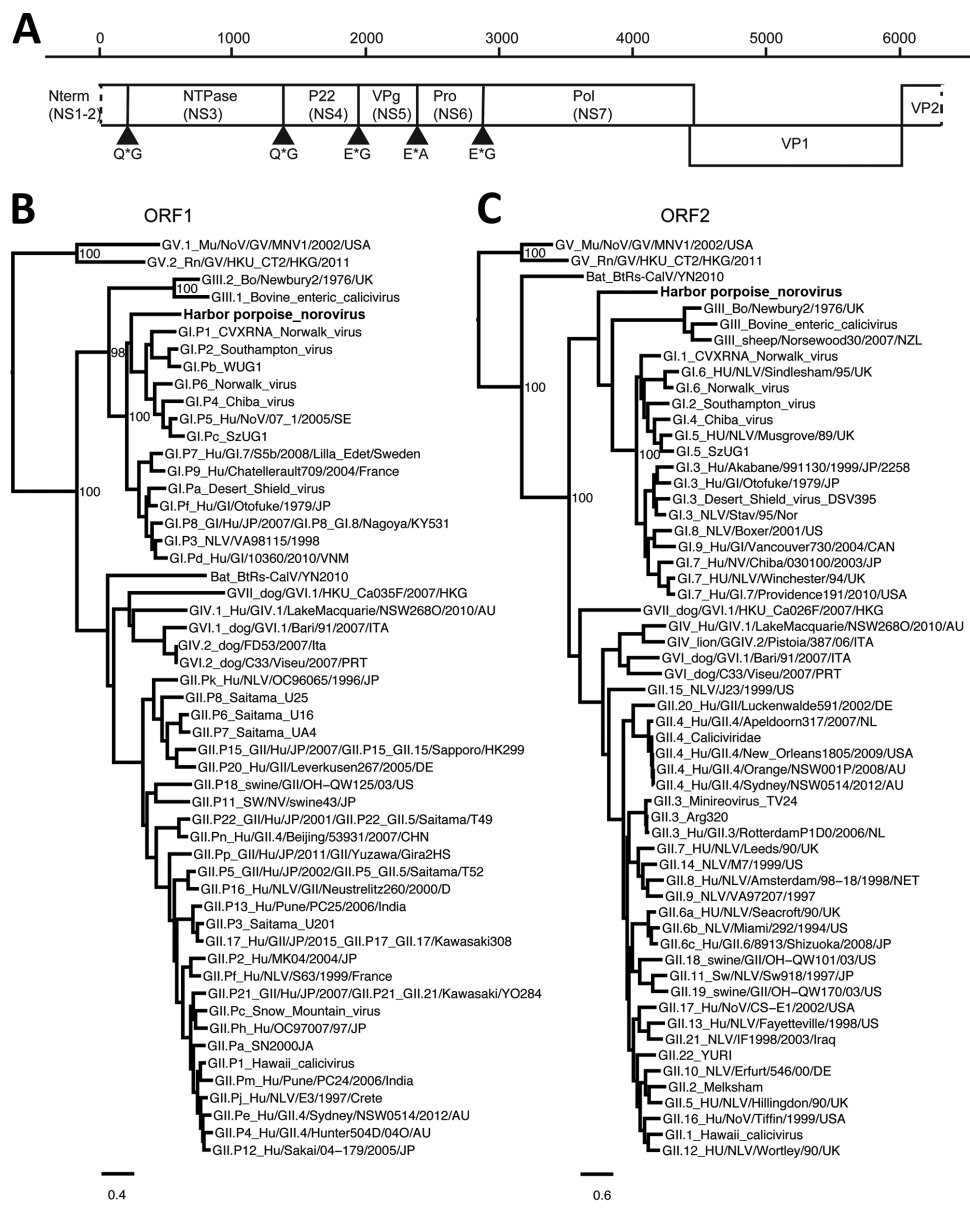
Genetic characterization of harbor porpoise norovirus. A) Genome organization of harbor porpoise norovirus. The putative cleavage sites are shown with arrowheads. B, C) Maximum-likelihood trees of the RNA-dependent RNA-polymerase (B) and ORF 2 (C) were inferred by PhyML 3.0 software (http://www.atgc-montpellier.fr/phyml/) by using the general time reversible nucleotide substitution model. Selected bootstrap values >70 are depicted. Scale bars indicate nucleotide substitutions per site. NS, nonstructural; NTPase, nucleoside triphosphatase; ORF, open reading frame; P, protein; Pol, polymerase; Pro, protease; VP, viral protein.

In the frame of a research program focusing on the identification of new viruses in possible reservoir hosts, we collected fecal material and performed random PCR in combination with 454-sequencing as described previously (*9*). This analysis resulted in 5,774 reads, of which 88 reads were most closely related to the norovirus genus, as determined by blastn and blastx analysis (*10*). Other reads that were most similar to viral genomes were most closely related to a coronavirus (16 reads [35%–94% nt identity]), salmon calicivirus (3 reads [11%–69% nt identity]), and porcine anello virus (1 read [91% nt identity], 2 reads [36% amino acid identity]). The harbor porpoise norovirus (HPNV) sequence was confirmed by Sanger sequencing (6,293 nt; GenBank accession no. KP987888) by using specific primers and comprising 3 ORFs, the partial ORF1 encoding the putative polyprotein, ORF2 encoding viral protein (VP) 1, and a partial ORF3 encoding VP2 (Figure 1, panel A). Phylogenetic analysis revealed that the HPNV RNA-dependent RNA-polymerase encoded by ORF1 clustered together with human genogroup I(GI) sequences (Figure 1, panel B), whereas VP1 clustered near strains belonging to human GI and bovine GIII (Figure 1, panel C).

To determine the tissue tropism of HPNV, we extracted RNA from formalin-fixed paraffin-embedded (FFPE) tissues collected from the HPNV-positive animal for histopathology. Tissues from all main organs and lymph nodes were tested for HPNV RNA by real-time PCR (Technical Appendix). Only the intestinal tissue and the FFPE material for immunohistochemical analysis (containing a mixture of tissues) were positive for norovirus with cycle threshold values of 30.5 and 37.4, respectively.

**Figure 2:**
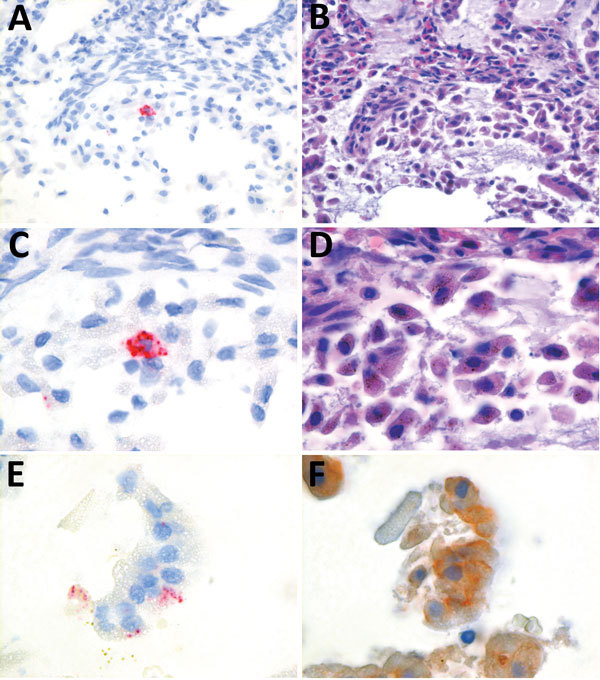
Detection of harbor porpoise norovirus transcripts in intestinal tissue of a harbor porpoise (*Phocoena phocoena*) using in situ hybridization with probes designed by Advanced Cell Diagnostics (Hayward, CA, USA), based on the 6,293 nt of harbor porpoise norovirus (A, C, E; original magnification ×40, ×100, ×100, respectively). Consecutive slides were stained with hematoxylin and eosin (B, D; original magnification ×40, ×100, respectively) and pankeratin (F, original magnification ×100), as described previously (*11*).

We conducted in situ hybridization to determine the cellular tropism of HPNV, as described previously (Figure 2) (*12*). HPNV-specific transcripts were detected in the cells in the intestine, indicating that this virus replicates in the intestinal tract. Sequential slides stained with hematoxylin and eosin or pankeratin showed that positive cells corresponded with enterocytes that had sloughed into the intestinal lumen because of freeze–thaw artifact, although we cannot exclude the possibility that other cell types were present (Figure 2).

To estimate the percentage of norovirus-infected harbor porpoises in our dataset, we extracted RNA from FFPE porpoise intestinal tissues collected from 48 animals during a 10-year period. Including the animal in which we detected HPNV, 10% (5/49) of the animals were positive for norovirus (cycle threshold <37) (Table 1), with primers designed to detect HPNV. Macroscopic and microscopic examination of the intestine of these animals showed no pathologic differences from harbor porpoises without norovirus infection.

**Table 1:** Formalin-fixed paraffin-embedded tissues subjected to reverse transcription PCR in a study of norovirus in 48 harbor porpoises (*Phocoena phocoena*), the Netherlands

Year	Tested samples, no.	Positive samples, no. (%)	Cycle threshold
2006	12	0	–
2007	4	1 (25)	33.8
2008	3	0	–
2009	1	0	–
2010	4	0	39.3
2011	10	1 (10)	33.8
2012	6	3 (50)	30.6, 36.8, 36.4
2013	4	0	–
2014	3	0	37.1
2015	2	0	–

To detect norovirus-specific antibodies in porpoise serum, we developed an ELISA based on HPNV VP1 and subsequently screened 34 harbor porpoise serum samples collected during 2006–2015 in the Netherlands (Technical Appendix). Samples from 8 (24%) harbor porpoises were positive for norovirus antibodies (Table 2). This dataset included samples from 2 harbor porpoises that were positive for norovirus RNA; however, their serum samples were negative for norovirus-specific antibodies.

**Table 2:** Prevalence of norovirus-specific antibodies in harbor porpoises (*Phocoena phocoena*), the Netherlands

Year	No. samples tested	Positive samples, no. (%)	ELISA titer
2006	12	1 (8)	>160
2007	4	1 (25)	20
2008	2	0	
2009	1	1 (100)	20
2010	5	2 (40)	40, 80
2011	5	3 (60)	20, 40, 40
2012	1	0	
2013	0	0	
2014	1	0	
2015	1	0	

## Conclusions

Similar to human noroviruses, HPNV replicates in the intestine. B cells and enterocytes support human norovirus replication in vitro (*13*,*14*). We detected HPNV in cells corresponding to enterocytes, and it will be interesting to determine whether these viruses share receptor use with other noroviruses. In humans, norovirus infections are self-limiting in healthy persons but can result in illness and death in high-risk groups (*4*). Because the harbor porpoise in which we detected HPNV did not exhibit clinical signs of gastrointestinal disease, norovirus infection probably was not a major factor in the death of this animal.

Remarkably, the HPNV displayed 99% sequence homology to a short (300-nt) norovirus VP1 sequence detected in oysters (*15*). These oysters had been sampled because they were associated with a foodborne gastroenteritis outbreak in Ireland in 2012 (*15*). Oyster samples were collected from the restaurant where the outbreak occurred and from their harvesting area. Strains belonging to genotypes GI.1, GI.4, GII.4, GII.3, GII.1, GII.6, GI.2, GII.7, GI.11, and the strain that was homologous to HPNV were detected, although the HPNV-like strain was detected only in oysters from the harvesting area. In the patients, only GI.4, GI.2, GI.6, GII.1, and GII.7 strains were detected, but the fact that noroviruses infecting marine mammals closely related to human noroviruses have been found infecting harbor porpoises and contaminating oysters raises the question of whether HPNV could infect humans through contamination of oysters or other shellfish.

On the basis of our findings that norovirus infections might be a common infection in harbor porpoises from the southern North Sea and the detection of a norovirus in a sea lion (*3*), it is not unlikely that noroviruses are common in other marine mammals as well. The high genetic diversity within this genus complicates detection of new noroviruses. The discovery of HPNV and the recent discovery of noroviruses in bats highlight that much still remains to be discovered about animal reservoirs of noroviruses and triggers questions about the zoonotic potential of these viruses.

Technical Appendix, Small cetaceans rescue and rehabilitation, norovirus RNA isolation and reverse transcription PCR, preparation of harbor porpoise norovirus major capsid protein, and ELISA to detect 52 harbor porpoise norovirus–specific antibodies.
