# Supplementary material for: Norovirus Infection in Harbor Porpoises
Source: Emerg Infect Dis. 2017 Jan;23(1):87–91. doi: 10.3201/eid2301.161081 (PMC5176230; doi:10.3201/eid2301.161081)
Supplement: Supplementary file 1 — Technical Appendix, Small cetaceans rescue and rehabilitation, norovirus RNA isolation and reverse transcription PCR, preparation of harbor porpoise norovirus major capsid protein, and ELISA to detect 52 harbor porpoise norovirus–specific antibodies. [file 16-1081-Techapp-s1.pdf]

Article DOI: http://dx.doi.org/10.3201/eid2301.161081

Norovirus Infection in Harbor Porpoises

Technical Appendix

Rescue and Rehabilitation of Small Cetaceans

Since 1967, small cetaceans—mainly harbor porpoises—that strand alive along the coasts of the Netherlands, Belgium, and Germany have been rehabilitated at the Dolfinarium Harderwijk (Harderwijk, the Netherlands) and subsequently released into the wild. Since 2004, this activity has been organized in the form of an independent foundation, SOS Dolphin which operates at the same site. The SOS Dolphin Foundation has two 50-m^3 pools with fresh water to which salt is added. In the first period of rehabilitation, animals are observed 24 hours per day, and standard parameters are recorded, including respiration rate, cramps, food intake, and defecation. In addition, other potentially relevant observations are recorded, including swimming behavior and alertness. As an animal improves, the level of observation and care diminish to a minimum of 9 hours per day. Admission and rehabilitation of live-stranded wild harbor porpoises at the SOS Dolphin Foundation was authorized by the government of the Netherlands (application no. FF/75/2012/036). The tissues and fecal material were collected during necropsies of stranded wild harbor porpoises, animals that died despite intensive care or were euthanized because of severe clinical signs in the absence of any indication of future recovery. The SOS Dolphin Foundation provided permission to the Department of Viroscience, Erasmus Medical Center (Rotterdam, the Netherlands), for the use of these samples for the present study.

RNA Isolation and Reverse Transcription PCR

Tissue samples were collected from harbor porpoises and stored in 10% (v/v) neutral buffered formalin solution, fixated for 1–2 days, and embedded in paraffin. For RNA isolation, 2 consecutive 10-µm thick tissue sections from intestinal tissues were collected from each porpoise. Nucleic acids are preserved for many years at room temperature in formalin-fixed paraffin-embedded tissues. Some degradation occurs, but it does not necessarily pose a problem for techniques that target short sequences, such as real-time PCR (RT-PCR) and 454 next-

generation sequencing (*1*). RNA was extracted using the RNAeasy formalin-fixed paraffin-embedded kit (QIAGEN, Hilden, Germany) according to manufacturer's instructions with a yield of 1.4–3.9 μg RNA. Harbor porpoise norovirus (HPNV) RNA was detected using RT-PCR. GI-specific primer sets and fluorescent probes in the open reading frame 1–2 junction region for use in RT-PCR (*2*) were adapted for the detection of HPNV. The primer pair CGYTGGATGCGNTTYCATGAC (forward) and AGTGGACGCCATCATCATTYAC (reverse) was used with a fluorescent probe AGATYGCGATCACCTGTCCA. RT-PCRs were performed by using a 7500 RT-PCR system (Applied Biosystems, Foster City, CA, USA) and Taqman Universal Mastermix (Applied Biosystems). The GI-specific primer set resulted in some amplification of HPNV but did not yield a sigmoidal curve. HPNV is not detected by genogroup II–specific primer probe sets (*3*,*4*).

Preparation of HPNV Major Capsid Protein

The HPNV major capsid protein (VP1) sequence was cloned into the mammalian expression vector pCAGGS by using EcoRI and XhoI sites. The pCAGGS-VP1 construct was transfected into 293T cells using the calcium phosphate method (*5*). After 3 days, the cells and media were collected. The cells were lysed as described previously (*6*). Next, the cell lysate and medium were centrifuged at 1,711 × g for 15 min at room temperature. Virus-like particles (VLPs) were pelleted by for spinning 2 h at 27,000 rpm and further purified by sucrose gradients. The purified VLPs were concentrated on centrifugal filters (Amicon, Darmstadt, Germany). Samples were subjected to sodium dodecyl sulfate polyacrylamide gel electrophoresis and stained with InstantBlue (Expedeon, San Diego, CA, USA) to confirm the expression of the VP1 protein (Technical Appendix Figure, panel A). The formation of VLPs was confirmed by electron microscopy (Technical Appendix Figure, panel B). VLPs were stored at −80°C until further use.

ELISA to Detect HPNV-Specific Antibodies

Plates (Costar, Corning, NY, USA) were coated overnight at 4°C with 10 ng/mL HPNV VLPs in phosphate-buffered saline (PBS), or as a negative control with cell-lysate from 239T cells transfected with empty pCAGGS. The following day, plates were washed 3 times with

PBS-Tween and blocked with 3% bovine serum albumin in PBS-Tween for 1 h at room temperature. Next, the wells were washed with PBS-Tween and incubated with 2-fold dilutions of porpoise serum at a starting dilution of 1:20 (in duplo). Immunoglobulins were detected by incubation with biotin-conjugated protein A (1 h at room temperature, Sigma-Aldrich, St. Louis, MO, USA), followed by an incubation step with streptavidine-horseradish peroxidase. After addition of the 3,3,5,5-tetramethylbenzidine substrate, the reaction was stopped by the addition of sulphuric acid solution (2 M) and the optical density was read at 450 nm. Some serum gave high background values; therefore, a serum sample was determined to be positive only if the optical density 450 values were equal or higher than twice the background of the same serum sample on negative control antigen.

References

1. Bodewes R, van Run PR, Schürch AC, Koopmans MP, Osterhaus AD, Baumgärtner W, et al. Virus characterization and discovery in formalin-fixed paraffin-embedded tissues. J Virol Methods. 2015;214:54–9. PubMed http://dx.doi.org/10.1016/j.jviromet.2015.02.002

2. Kageyama T, Kojima S, Shinohara M, Uchida K, Fukushi S, Hoshino FB, et al. Broadly reactive and highly sensitive assay for Norwalk-like viruses based on real-time quantitative reverse transcription-PCR. J Clin Microbiol. 2003;41:1548–57. PubMed http://dx.doi.org/10.1128/JCM.41.4.1548-1557.2003

3. Butot S, Le Guyader FS, Krol J, Putallaz T, Amoroso R, Sánchez G. Evaluation of various real-time RT-PCR assays for the detection and quantitation of human norovirus. J Virol Methods. 2010;167:90–4. PubMed http://dx.doi.org/10.1016/j.jviromet.2010.03.018

4. Le Guyader FS, Parnaudeau S, Schaeffer J, Bosch A, Loisy F, Pommepuy M, et al. Detection and quantification of noroviruses in shellfish. Appl Environ Microbiol. 2009;75:618–24. PubMed http://dx.doi.org/10.1128/AEM.01507-08

5. de Wit E, Spronken MI, Bestebroer TM, Rimmelzwaan GF, Osterhaus AD, Fouchier RA. Efficient generation and growth of influenza virus A/PR/8/34 from eight cDNA fragments. Virus Res. 2004;103:155–61. PubMed http://dx.doi.org/10.1016/j.virusres.2004.02.028

6. Debbink K, Costantini V, Swanstrom J, Agnihothram S, Vinje J, Baric R, et al. Human norovirus detection and production, quantification, and storage of virus-like particles. Curr Protoc Microbiol. 2013;31:15K.1.1-15K.1.45. **PMID: 24510290**

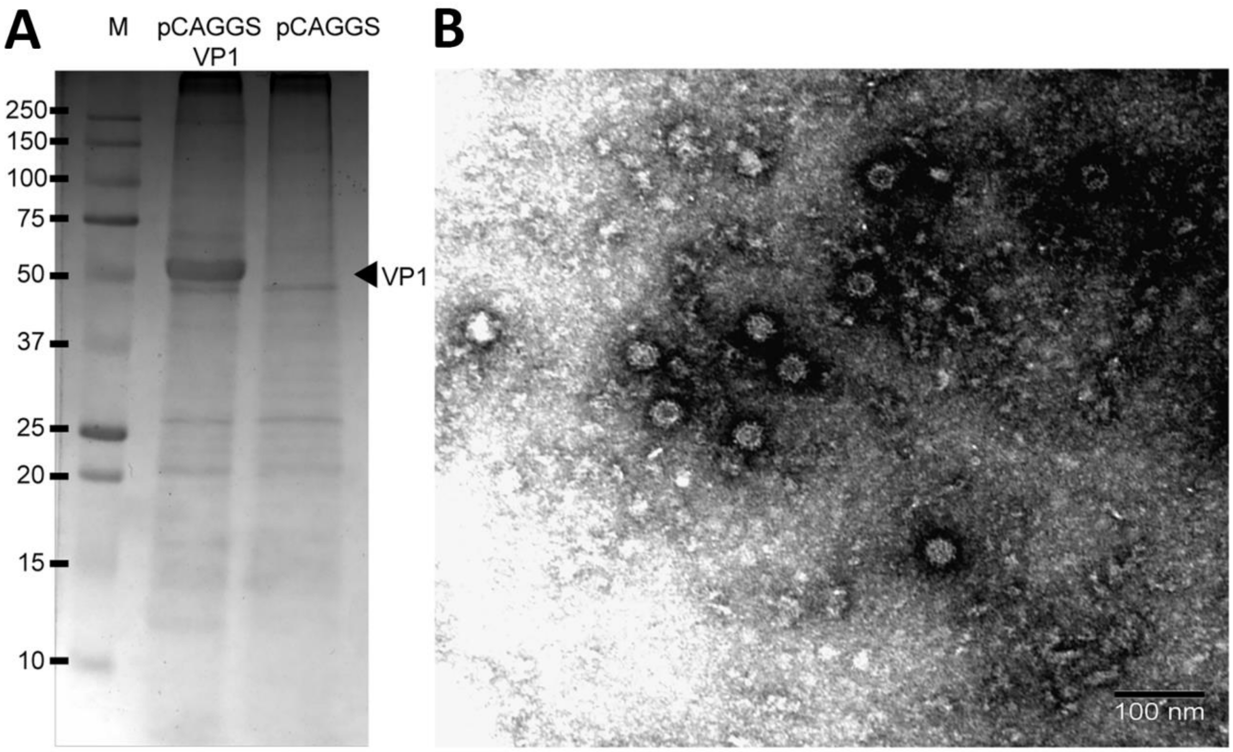

Technical Appendix Figure. Sodium dodecyl sulfate polyacrylamide gel electrophoresis gel with marker (lane 1), VLPs isolated from 293T cells transfected with pCAGGS-VP1 (lane 2) and 293T cells transfected with empty pCAGGS (lane 3) to confirm the expression of harbor porpoise norovirus VP1 (A). Analysis of VLPs by electron microscopy (B). VLP, virus-like particles; VP, viral protein.
